# AlzGPS: a genome-wide positioning systems platform to catalyze multi-omics for Alzheimer’s drug discovery

**DOI:** 10.1186/s13195-020-00760-w

**Published:** 2021-01-13

**Authors:** Yadi Zhou, Jiansong Fang, Lynn M. Bekris, Young Heon Kim, Andrew A. Pieper, James B. Leverenz, Jeffrey Cummings, Feixiong Cheng

**Affiliations:** 1 Genomic Medicine Institute, Lerner Research Institute, Cleveland Clinic, Cleveland, OH 44195 USA; 2 Department of Molecular Medicine, Cleveland Clinic Lerner College of Medicine, Case Western Reserve University, Cleveland, OH 44195 USA; 3 Harrington Discovery Institute, University Hospitals Cleveland Medical Center, Cleveland, OH 44106 USA; 4 Department of Psychiatry, Case Western Reserve University, Cleveland, OH 44106 USA; 5 Geriatric Psychiatry, GRECC, Louis Stokes Cleveland VA Medical Center, Cleveland, OH 44106 USA; 6 Institute for Transformative Molecular Medicine, School of Medicine, Case Western Reserve University, Cleveland, OH 44106 USA; 7 Weill Cornell Autism Research Program, Weill Cornell Medicine of Cornell University, New York, NY 10065 USA; 8 Department of Neuroscience, School of Medicine, Case Western Reserve University, Cleveland, OH 44106 USA; 9 Lou Ruvo Center for Brain Health, Neurological Institute, Cleveland Clinic, Cleveland, OH 44195 USA; 10 Cleveland Clinic Lou Ruvo Center for Brain Health, Las Vegas, NV 89106 USA; 11 Chambers-Grundy Center for Transformative Neuroscience, Department of Brain Health, School of Integrated Health Sciences, UNLV, Las Vegas, NV 89154 USA; 12 Case Comprehensive Cancer Center, School of Medicine, Case Western Reserve University, Cleveland, OH 44106 USA

**Keywords:** Alzheimer’s disease, Clinical trial, Drug repurposing, Genomics, Mechanism-of-action, Multi-omics, Network medicine, Systems pharmacology

## Abstract

**Background:**

Recent DNA/RNA sequencing and other multi-omics technologies have advanced the understanding of the biology and pathophysiology of AD, yet there is still a lack of disease-modifying treatments for AD. A new approach to integration of the genome, transcriptome, proteome, and human interactome in the drug discovery and development process is essential for this endeavor.

**Methods:**

In this study, we developed AlzGPS (*G*enome-wide *P*ositioning *S*ystems platform for *Alz*heimer’s Drug Discovery, https://alzgps.lerner.ccf.org), a comprehensive systems biology tool to enable searching, visualizing, and analyzing multi-omics, various types of heterogeneous biological networks, and clinical databases for target identification and development of effective prevention and treatment for AD.

**Results:**

Via AlzGPS: (1) we curated more than 100 AD multi-omics data sets capturing DNA, RNA, protein, and small molecule profiles underlying AD pathogenesis (e.g., early vs. late stage and tau or amyloid endophenotype); (2) we constructed endophenotype disease modules by incorporating multi-omics findings and human protein-protein interactome networks; (3) we provided possible treatment information from ~ 3000 FDA approved/investigational drugs for AD using state-of-the-art network proximity analyses; (4) we curated nearly 300 literature references for high-confidence drug candidates; (5) we included information from over 1000 AD clinical trials noting drug’s mechanisms-of-action and primary drug targets, and linking them to our integrated multi-omics view for targets and network analysis results for the drugs; (6) we implemented a highly interactive web interface for database browsing and network visualization.

**Conclusions:**

Network visualization enabled by AlzGPS includes brain-specific neighborhood networks for genes-of-interest, endophenotype disease module networks for omics-of-interest, and mechanism-of-action networks for drugs targeting disease modules. By virtue of combining systems pharmacology and network-based integrative analysis of multi-omics data, AlzGPS offers actionable systems biology tools for accelerating therapeutic development in AD.

**Supplementary Information:**

The online version contains supplementary material available at 10.1186/s13195-020-00760-w.

## Background

Alzheimer’s disease (AD) is a progressive neurodegenerative disorder accounting for 60–80% of dementia cases [1]. In addition to cognitive decline, AD patients have extensive neuropathological changes including deposition of extracellular amyloid plaques, intracellular neurofibrillary tangles, and neuronal death [2, 3]. It is estimated that the number of AD patients will reach 16 million by 2050 in the USA alone [4, 5]. Effective treatments are needed, as there are no disease-modifying treatments for AD and no new drugs have been approved since 2003 by the US Food and Drug Administration (FDA). There are several possible explanations for the high failure rate in AD drug discovery. For example, traditional “one gene, one drug, one disease” hypothesis may result in failure by anticipated off-target side effects and suboptimal efficacy because of complex disease pathobiology of AD [3, 6]. Also, there is a lack of sensitive endpoint measures for outcomes in clinical trials. Other potential immediate causes for clinical trial failures include targeting the wrong pathobiological or pathophysiological mechanisms, attempted intervention at the wrong stage (too early or too late), unfavorable pharmacodynamic and pharmacokinetic characteristics of the drug (e.g., poor brain penetration), lack of target engagement by drug candidates, and hypotheses that fail to incorporate the great complexity of AD [6, 7].

**Fig. 1:**
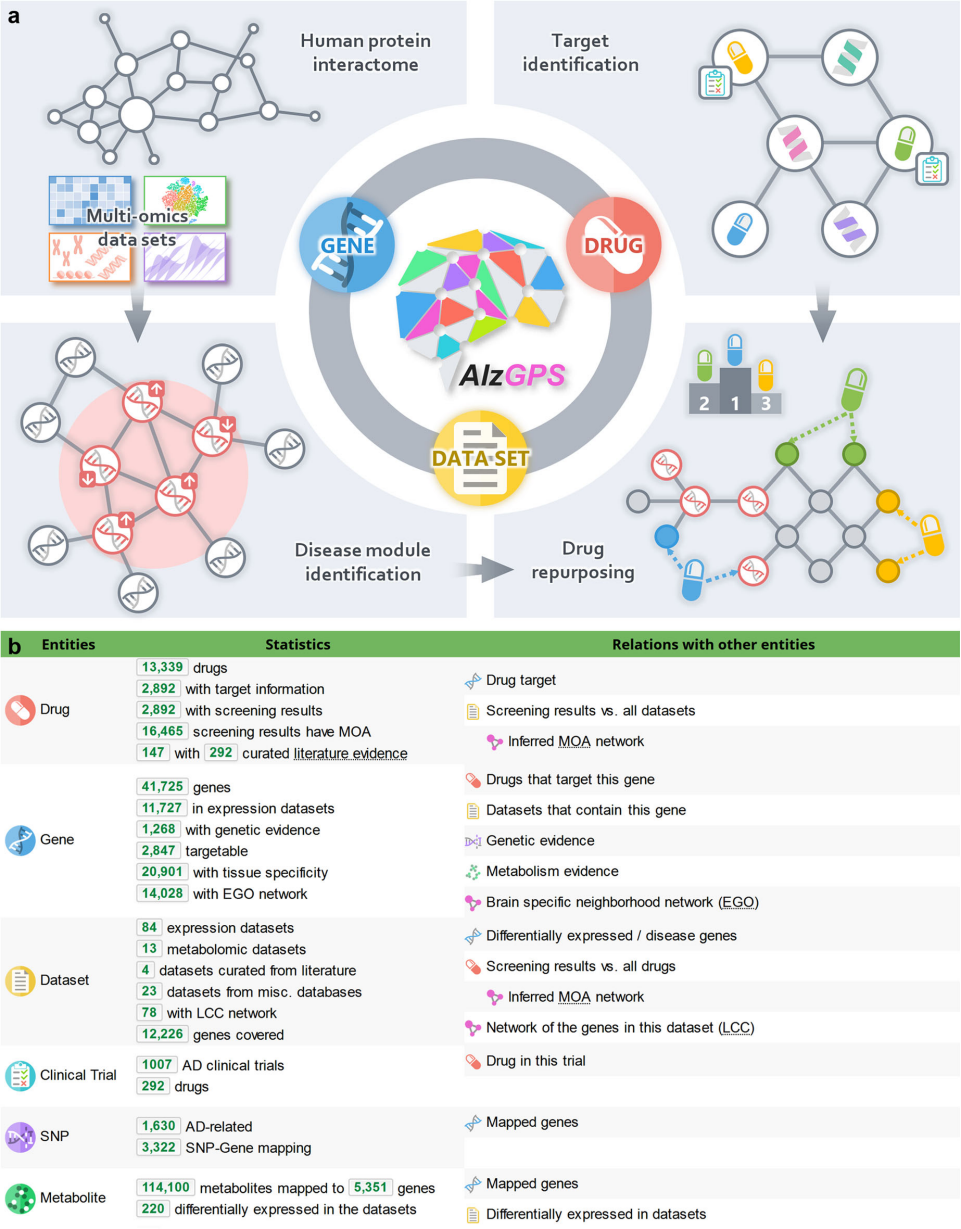
The architecture of AlzGPS. **a** AlzGPS was built on three main data entities (genes, drugs, and omics layers) and their relationships. The multi-omics data (genomics, transcriptomics (bulk and single cell/single nucleus), and proteomics) in AlzGPS help identify likely causal genes/targets that are associated with Alzheimer’s disease (AD) and disease modules in the context of human protein-protein interactome. Via network proximity measure between drug-target networks and disease modules in the human protein-protein interactome, drugs can be prioritized for their potential to alter the genes in the module for potential treatment of AD. **b** Detailed statistics of the entities and relations in AlzGPS. EGO, brain-specific neighborhood network (ego network); LCC, largest connected component network; MOA, mechanism-of-action network

Multiple types of omics data have greatly facilitated our understanding of the pathobiology of AD. For example, using single-cell RNA-Seq, a novel microglia type (termed disease-associated microglia, DAM) was discovered to be associated with AD, understanding of whose molecular mechanism could offer new therapeutic targets [8]. Using large-scale genome-wide association studies (GWAS), twenty loci showed genome-wide significant association with AD, among which 11 were newly discovered [9]. A recent study using deep profiling of proteome and phosphoproteome prioritized proteins and pathways associated with AD, and it was shown that protein changes and their corresponding RNA levels only partially coincide [10]. The large amount of multi-omics data and recent advances in network-based methodologies for drug repurposing today present unprecedented opportunities for accelerating target identification for drug discovery for AD. This potential has been demonstrated in other complex diseases as well, such as cancer [11], cardiovascular disease [12], and schizophrenia [13], and is beginning to be exploited in AD [6, 14]. Drug repurposing offers a rapid and cost-effective solution for drug discovery for complex disease, such as the current global pandemic of coronavirus disease 2019 (COVID-19) [15, 16] and AD [6]. The central idea of network-based drug repurposing is that for a drug to be able to affect a disease, the drug targets must directly overlap with or be in the immediate vicinity of the disease modules, which can be identified using the vast amount of high-throughput multi-omics data (Fig. 1a). Our recent efforts using network-based methodologies and AD omics data have led to the discovery of two drugs that show efficacy in network models in AD: sildenafil [6] and pioglitazone [14]. Network analysis provides potential mechanisms for these drugs and facilitates experimental validation. Therefore, we posit that a comprehensive systems biology tool in the framework of network-based multi-omics analysis could inform Alzheimer’s patient care and therapeutic development.

To this end, we present a new freely available database and tool, named AlzGPS (A *G*enome-wide *P*ositioning *S*ystems platform for *Alz*heimer’s Drug Discovery), for target identification and drug repurposing for AD. AlzGPS was built with large-scale diverse information, including multi-omics (genomics, transcriptomics [bulk and single cell], proteomics, and interactomics) of human and other species, drug-target networks, literature-derived evidence, AD clinical trials information, and network proximity analysis (Fig. 1b). Our hope is that AlzGPS will be a valuable resource for the AD research community for several reasons. First, AlzGPS contains abundant multi-domain information all coalesced in one location. The manually curated data, such as the literature-derived information for the most promising repurposable drugs and more than 100 multi-omics AD data sets, are of high quality and relevance. Second, using state-of-the-art network proximity approaches, AlzGPS provides a systemic evaluation of 3000 FDA approved or investigational drugs against the AD data sets. These results (along with various network visualizations) will provide insights for potential repurposable drugs with clear network-based footprints in the context of the human protein-protein interactome. The drug-data set associations can be further explored in AlzGPS for individual drug targets or genes associated with AD. Lastly, AlzGPS offers a highly interactive and intuitive modern web interface. The relational nature of these data was embedded in the design to help the user easily navigate through different types of information. In addition, AlzGPS provides three types of network visualizations for the tens of thousands of networks in the database, including brain-specific neighbor networks for genes, disease modules derived from multi-omic profiles with varying degrees of disease biology of AD, and inferred mechanism-of-action (MOA) networks for drugs and omic pairs with significant proximity. AlzGPS is freely available to the public without registration requirement at https://alzgps.lerner.ccf.org.

## Methods

### Data collection and preprocessing

#### AD data sets

A data set is defined as either (1) genes/proteins/metabolites that are differentially expressed in AD patients/mice vs. controls, or (2) genes that have known associations with risks of AD from literature or other databases. We retrieved expression data sets underlying AD pathogenesis capturing transcriptomics (microarray, bulk or single-cell RNA-Seq) and proteomics across human, mouse, and model organisms (e.g., fruit fly and *Caenorhabditis elegans*). All the samples of the data sets were derived from total brain, specific brain regions (including hippocampus, cortex, and cerebellum), and brain-derived single cells, such as microglial cells. For some of the expression data sets, the differentially expressed genes/proteins were obtained from the original publications (from main tables or supplemental tables). For other data sets that did not have such differential expression results available, the original brain microarray/RNA-Seq data were obtained from Gene Expression Omnibus (GEO) [17] and differential expression analysis was performed using the tool GEO2R [18]. GEO2R performs the differential expression analysis for the sample groups defined by the user using the limma R package [19]. All differentially expressed genes identified in mouse were further mapped to unique human-orthologous genes using the NCBI HomoloGene database (https://www.ncbi.nlm.nih.gov/homologene). The details for all the data sets, including organism, genetic model (for mouse), brain region, cell type (for single-cell RNA-Seq), PubMed ID, GEO ID, and the sources (e.g., supplemental table or GEO2R), can be found in Table S1.

#### Genes and proteins

We retrieved the gene information from the HUGO Gene Nomenclature Committee (HGNC, https://www.genenames.org/) [20], including gene symbol, name, type (e.g., coding and non-coding), chromosome, synonyms, and identification (ID) mapping in various other databases such as NCBI Gene, ENSEMBL, and UniProt. All proteins from the AD proteomics data sets were mapped to genes using the mapping information from HGNC.

#### Single-nucleotide polymorphisms (SNPs)

We found 3321 AD-associated genetic records for 1268 genes mapped to 1629 SNPs, by combining results from GWAS Catalog (https://www.ebi.ac.uk/gwas/) [21] using the trait “Alzheimer’s disease” and published studies. The PubMed IDs for the genetic evidence are provided in AlzGPS.

#### Tissue expression specificity

We downloaded RNA-Seq data (transcripts per million, TPM) across 33 human tissues from the GTEx v8 release (accessed on March 31, 2020, https://gtexportal.org/home/). We defined the genes with count per million (CPM) ≥ 0.5 in over 90% samples (e.g., brain) as tissue-expressed genes and otherwise as tissue-unexpressed. To quantify the expression significance of tissue-expressed gene *i* in tissue *t*, we calculated the average expression 〈*E*(*i*)〉 and the standard deviation *δ*_*E*_(*i*) of a gene’s expression across all included tissues. The significance of gene expression in tissue *t* is defined as:

1 $$ {z}_E\left(i,t\right)=\frac{E\left(i,t\right)-\left\langle E(i)\right\rangle }{\delta_E(i)} $$

Data for multiple brain regions were available from GTEx v8. We combined the data of these brain regions when comparing the brain expression specificity vs. other tissues. In addition, we further computed the expression specificity across 13 different brain regions. Both tissue expression specificity and brain region expression specificity results for the genes are available in AlzGPS.

#### Drugs

We retrieved drug information from the DrugBank database (v4.3) [22], including name, type, group (approved, investigational, etc.), Simplified Molecular-Input Line Entry System (SMILES), and Anatomical Therapeutic Chemical (ATC) code(s). We also evaluated the pharmacokinetic properties (such as blood–brain barrier [BBB]) of the drugs using admetSAR [23, 24].

#### Drug literature information for AD treatment

For the top 300 repurposable drugs (i.e., drugs with the highest number of significant proximities to the AD data sets), we manually searched and curated the literature for their therapeutic efficacy against AD using PubMed. In addition to the title, journal, and PubMed ID, we summarized the types (clinical and non-clinical), experimental settings (e.g., mouse/human and transgenic line for non-clinical studies; patient groups, randomization type, length, and control type of clinical studies), and results of these studies. In total, we found 292 studies for 147 drugs.

#### Drug-target network

To build a high-quality drug-target network, several databases were accessed, including the DrugBank database (v4.3) [22], Therapeutic Target Database (TTD) [25], PharmGKB database, ChEMBL (v20) [26], BindingDB [27], and IUPHAR/BPS Guide to PHARMACOLOGY [28]. Only biophysical drug-target interactions involving human proteins were included. To ensure data quality, we kept only interactions that have inhibition constant/potency (K_i_), dissociation constant (K_d_), median effective concentration (EC_50_), or median inhibitory concentration (IC_50_) ≤ 10 μM. The final drug-target network contains 21,965 interactions among 2892 drugs and 2847 human targets/proteins.

#### Clinical trials

The AD interventional clinical trials were retrieved from https://clinicaltrials.gov. Information including phase, posted date, status, and agent(s) was obtained from https://clinicaltrials.gov. Drugs were mapped to the DrugBank IDs. Proposed mechanism and therapeutic purpose were from Cummings et al. [29, 30].

#### Human protein interactome

We used our previously built high-quality comprehensive human protein interactome which contains 351,444 unique protein-protein interactions (PPIs, edges) among 17,706 proteins (nodes) [11, 12, 31, 32]. Briefly, five types of evidence were considered for building the interactome: physical PPIs from protein three-dimensional (3D) structures, binary PPIs revealed by high-throughput yeast-two-hybrid (Y2H) systems, kinase-substrate interactions by literature-derived low-throughput or high-throughput experiments, signaling networks by literature-derived low-throughput experiments, and literature-curated PPIs identified by affinity purification followed by mass spectrometry (AP-MS), Y2H, or by literature-derived low-throughput experiments. The details are provided in our previous studies [11, 12, 31, 32].

### Network proximity quantification of drugs and AD data sets

To quantify the associations between drugs and AD-related gene sets from the data sets, we adopted the “closest” network proximity measure:

2 $$ \left\langle {d}_{AB}\right\rangle =\frac{1}{\left|\left|A\right|\right|+\left\Vert B\right\Vert}\left(\sum \limits_{a\in A}{\min}_{b\in B}d\left(a,b\right)+\sum \limits_{b\in B}{\min}_{a\in A}d\left(a,b\right)\right) $$

where *d*(*a*, *b*) is the shortest path length between gene *a* and *b* from gene list *A* (drug targets) and *B* (AD genes), respectively. To evaluate whether such proximity was significant, we performed *z* score normalization using a permutation test of 1000 random experiments. In each random experiment, two randomly generated gene lists that have similar degree distributions to *A* and *B* were measured for the proximity. The *z* score was calculated as:

3 $$ {z}_d=\frac{d-\overline{d}}{\sigma_d} $$

*P* value was calculated according to the permutation test. Drug-data set pairs with *Z* < − 1.5 and *P* < 0.05 were considered significantly proximal. In addition to network proximity, we calculated two additional metrics, overlap coefficient *C* and Jaccard index *J*, to quantify the overlap and similarity of *A* and *B*:

4 $$ C=\frac{\left|A\cap B\right|}{\min \left(\left|A\right|,\left|B\right|\right)} $$

5 $$ J=\frac{\left|A\cap B\right|}{\left|A\cup B\right|} $$

### Generation of gene/protein networks

We offer three types of networks in AlzGPS: brain-specific neighborhood (EGO) network for the genes, largest connected component (LCC) network for the data sets, and inferred MOA network for significantly proximal drug-data set pairs. The three networks differ by inclusion criteria of the nodes (genes/proteins). The edges are PPIs colored by their types (e.g., 3D, Y2H, and literature). All networks are colored by whether they can be targeted by the drugs in our database.

For the EGO networks, we filtered genes by their brain expression and generated only the network for those that were considered to be expressed in brain using GTEx data. We used the *ego_graph* function from NetworkX [33] to generate the EGO networks. The networks are centered around the genes-of-interest. We incorporated the tissue specificity of the genes (indicated in the network by the node size) into the visualization tool, to allow users to further filter the network to show only the genes that have positive brain specificity.

An LCC network was generated for each AD data set using the *subgraph* function from networkx. For MOA, we examined the connections (PPIs) among the drug targets and the data sets.

### Website implementation

AlzGPS was implemented with the Django v3.1.0 framework (www.djangoproject.com). The website frontend was implemented with HTML, CSS, and JavaScript. The frontend was designed to be highly interactive and integrative. It uses AJAX to asynchronously acquire data in JSON format based on user requests to dynamically update the frontend interface. This architecture can therefore be integrated into end users’ own pipelines. Network visualizations were implemented using Cytoscape.js [34].

## Results

### Information architecture and statistics

One key feature of AlzGPS is the highly diverse yet interconnected data types (Fig. 1). The three main data types are genes, drugs, and AD-relevant omics data sets. More than 100 omics data sets were processed, including 84 expression data sets (Table S1) from AD transgenic animal models or patient-derived samples, 27 data sets from the literature or from other databases, and 13 metabolomic data sets. The expression data sets contain transcriptomic and proteomic data of human and rodent samples. Comparative sample groups were available in these data sets, such as early stage vs. late stage, healthy vs. AD. The differentially expressed genes/proteins were calculated for each data set.

The statistics and relations of the database are shown in Fig. 1b. We collected and processed all the basic information (see the “Methods” section) and then constructed the relationships among the data types. For example, for genes and drugs, the relationship is drugs targeting proteins (genes); for genes and data sets, the relationship is genes being differentially expressed in the expression data sets or included in other types of data sets, such as literature-based; for drugs and data sets, the proximity between each pair was calculated (see the “Methods” section) to identify the drug that is significantly proximal to a data set, and vice versa.

Additional data types were collected or generated. For genes, these included genetic evidence (variants associated with AD) and tissue expression specificity to provide additional information for target gene identification. For drugs, we collected the data from more than 1000 AD clinical trials, and included the proposed mechanisms-of-action and possible therapeutic indications on AD [29, 30]. Drugs of these trials were extracted such that users can open associated drugs from the trial page. The BBB probability was computed [23, 24], as well as 23 other predicted absorption, distribution, metabolism, excretion, and toxicity (ADMET) properties. For the top 300 drugs with the highest number of significant proximities to all the data sets, we manually curated the available literature. A total of 292 studies were found for 147 drugs (49%) that reported the associations of the drugs and AD. We grouped these studies into clinical and non-clinical, and extracted trial information for clinical type and experimental setting (number and type of patients) for both types. We also summarized and provided the study results.

### Web interface and network visualizations

**Fig. 2:**
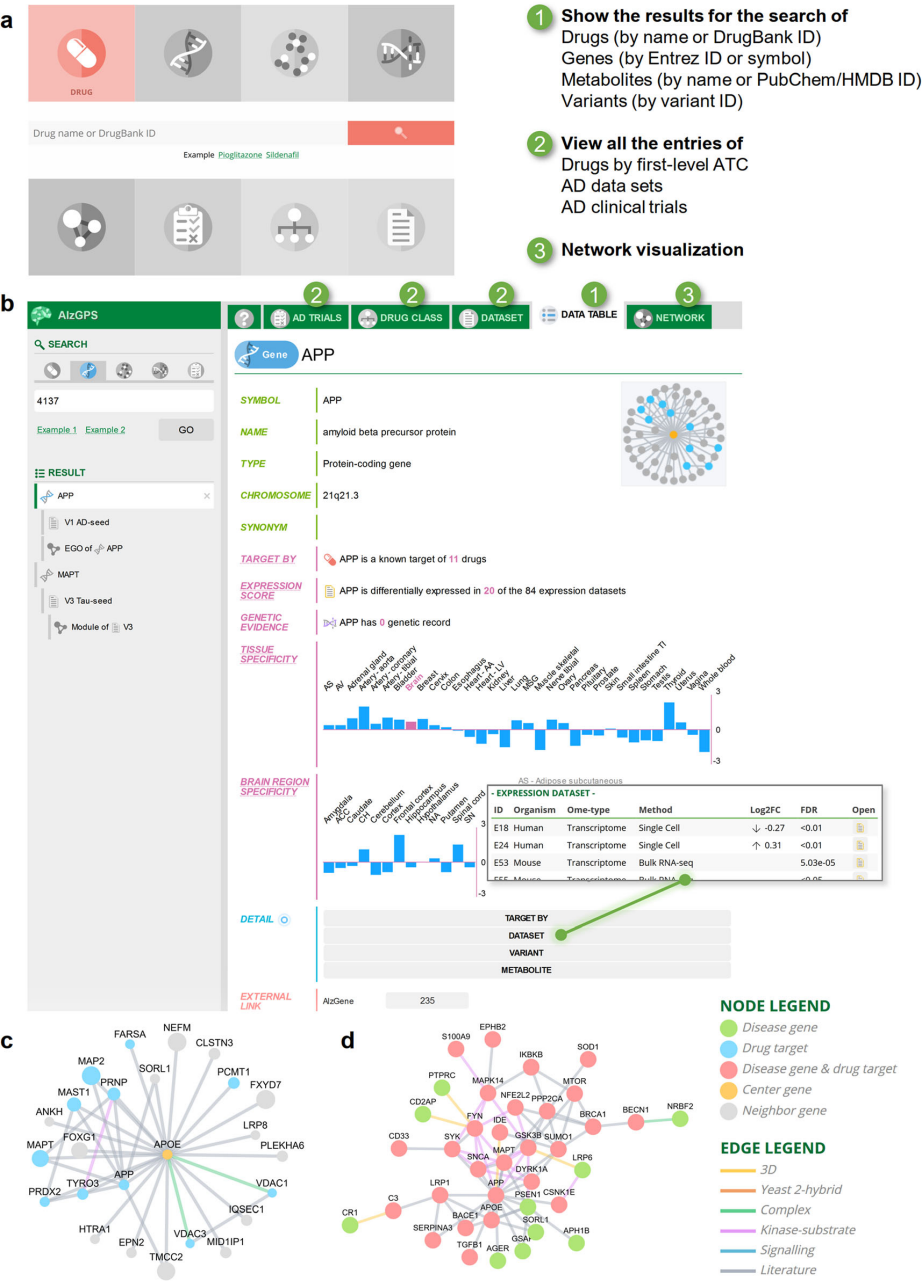
Web interface overview. **a** The home page provides access to searching, listing entries, and viewing brain-specific gene/target networks. User will be redirected to the interactive explorer (**b**), in which all information is then dynamically loaded and added to the same web page. Each data entity has its own basic information page under the “DATA TABLE” tab. Additional information regarding the relations (e.g., proximity results) can be loaded by clicking the corresponding button in the “DETAIL” section. **c** An example brain-specific neighborhood network using APOE. **d** An example largest connected component network using data set “V2”

A highly interactive web interface was implemented (Fig. 2). On the home page (Fig. 2a), the user can search for drugs, genes, metabolites, gene variants, and clinical trials. The user can directly list all drugs by their first-level ATC code, all AD data sets available, and all the AD clinical trials (Fig. 2b). The search results are displayed in the “DATA TABLE” tab and switched with their associated buttons in the “RESULT” section on the left. Each data entity has its own data table for the associated information in the “DATA TABLE” tab. For example, on the gene page of APP (Fig. 2b) is the basic information (green rows), such as name, type, chromosome, and synonym; descriptions for the derived data (purple rows), such as tissue specificity and number of genetic records; and external links (red row). Data for the relations of APP and other entities can be loaded by clicking the button in “DETAIL” (blue row). For example, the expression data sets in which APP is differentially expressed can be found by clicking the “DATASET” button (Fig. 2b). Any data loaded will be added to the same explorer. The buttons in the “RESULT” are organized in trees. For example, APP is included in the “V1 AD-seed” data set, which contains 144 AD-associated genes with strong literature evidence. When the user clicks this data set in the APP gene table, a new data table for the “V1 AD-seed” data set will replace the APP gene page, and a new button with indentation will appear below the APP button in “RESULT” (Fig. 2b).

**Fig. 3:**
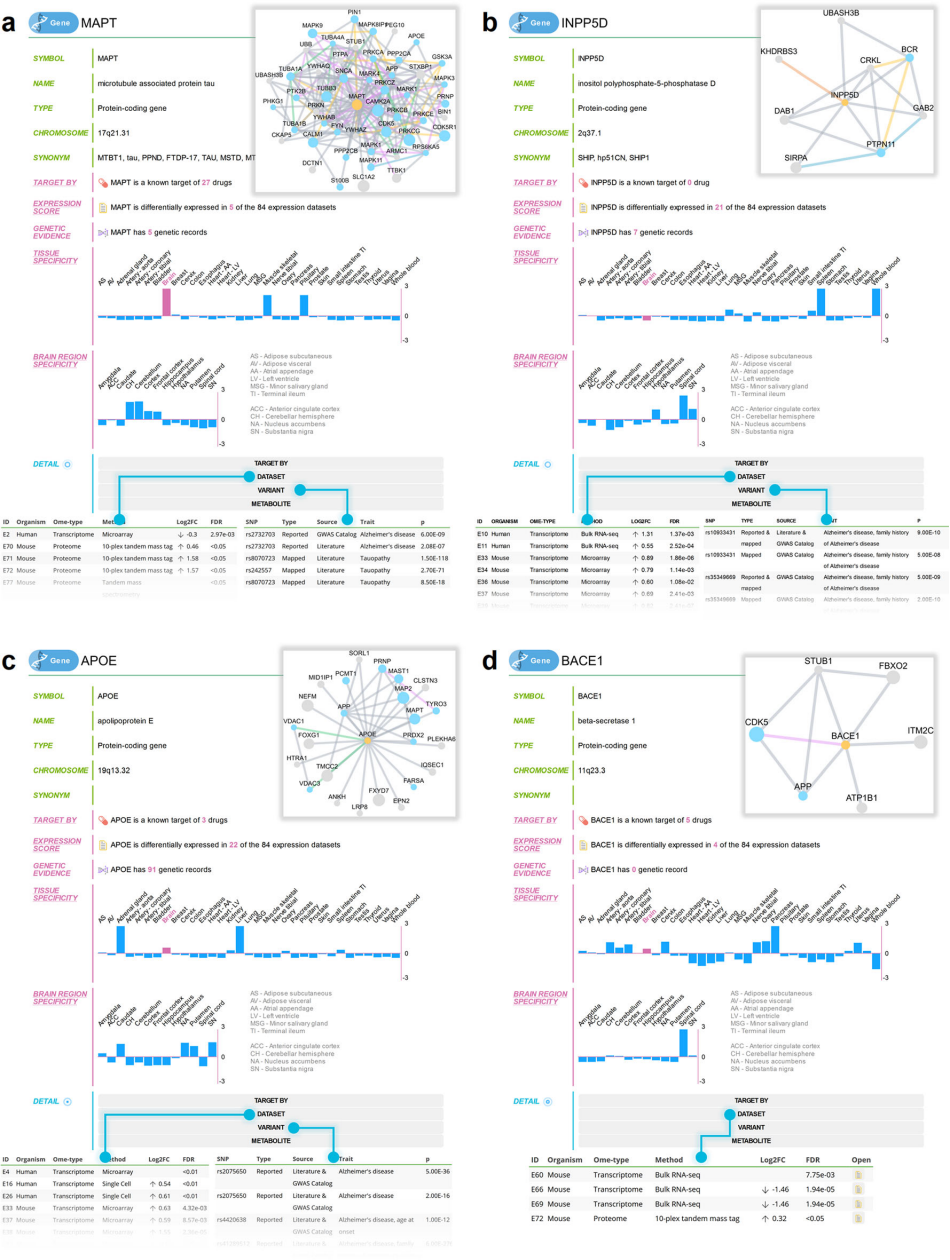
Case study—target identification. Four genes, MAPT (**a**), INPP5D (**b**), APOE (**c**), and BACE1 (**d**), are used as examples to show the gene page. On the gene page, we show a summary of several statistics of the gene in AlzGPS, including the number of drugs that can target it, number of data sets of omics in which the target/protein coding gene is differentially expressed, number of genetic records, and the brain expression specificity. Detailed information can be loaded by clicking corresponding buttons. Examples of detailed differential expression results and genetic records are shown for these four genes. In addition, a brain-specific neighborhood network is available that centers around the gene-of-interest and shows the targetability of its neighborhood

The all-in-one interactive explorer that minimizes the need for navigation of information using the relational nature of these data is a major feature of the web interface. Another major feature is the network visualizations. We offer three types of networks: (1) the brain-specific neighborhood network (EGO) for a gene-of-interest that shows the PPIs with its neighbors (Fig. 2c), (2) the largest connected component (LCC) network for a data set that shows the largest module formed by the genes in this data set (Fig. 2d), and (3) inferred MOA network for a significantly proximal drug-data set pair, which is illustrated in the case studies below.

### Case study—target identification

Generally, using AlzGPS for AD target identification starts with selecting one or a set of data sets (Fig. 2b, “DATASET” tab). Users can select a data set based on organisms, methods (e.g., single-cell/nuclei RNA-Seq), brain regions, and comparisons (e.g., early-onset AD vs. healthy control) for the expression data sets. Additionally, we have collected data sets from the literature, other databases, or computationally predicted results. Here, we use the “V1 AD-seed” data set as a starting point. This data set was from our recent study which contains 144 AD-associated genes based on literature-derived evidence. We found that 118 genes were differentially expressed as shown in at least one data set. By browsing these genes, we selected four examples, microtubule-associated protein tau (MAPT), inositol polyphosphate-5-phosphatase D (INPP5D), apolipoprotein E (APOE), and β-secretase 1 (BACE1) based on positive brain expression specificity and number of data sets that include them.

#### MAPT

MAPT encodes the tau protein, modification of which is one of the main neuropathological hallmarks of AD [35, 36]. Mutations and alternative splicing of MAPT are associated with risk of AD [37]. MAPT is differentially expressed in five expression data sets (Fig. 3a) and has high brain specificity. Five pieces of genetic evidence were found for MAPT. MAPT can be targeted by 27 drugs. In addition, many of its direct PPI neighbors are targetable, suggesting a potential treatment strategy by targeting MAPT and its neighbors.

#### INPP5D

We found 7 genetic association records for INPP5D (Fig. 3b). Recent GWAS results showed that the rs35349669 polymorphism of INPP5D was significantly associated with an increased risk of late-onset AD in Caucasians [9, 38]. The intronic SNP rs61068452 of INPP5D was significantly associated with reduced cerebrospinal fluid (CSF) t-tau/Aβ_1–42_ ratio, showing a potentially protective role in AD [39]. In addition to these genetic associations, INPP5D was also differentially manifested across 21 human and mouse expression data sets. Altogether, INPP5D may suggest potential drug target candidates for future therapeutic development.

#### APOE

APOE has three major alleles, ε2, ε3, and ε4. Individuals carrying the ε4 allele have an increased risk of developing AD compared to those carrying the more common ε3 allele, while ε2 decreases the risk [40, 41]. The ε4 allele of APOE is the main genetic risk factor of AD [41]. APOE ε4 plays an important role in Aβ deposition [41], a major pathological hallmark of AD. APOE is differentially expressed in 22 data sets (Fig. 3c). It has a high number of associated genetic records—91. Both APOE and its PPI partners can be targeted.

#### BACE1

BACE1 cleaves APP and generates Aβ peptides [42], whose aggregation is a pathological hallmark of AD. The inhibition of BACE1 has been a popular target for AD drug development. Shown in Fig. 3d, BACE1 is differentially expressed in 4 data sets.

### Case study—drug repurposing

In this section, we use sildenafil and pioglitazone as two examples. In our recent studies, we found that both sildenafil and pioglitazone were associated with a reduced risk of AD using network proximity analysis and retrospective case-control validation [14]. Mechanistically, in vitro assays showed that both drugs were able to downregulate cyclin-dependent kinase 5 (CDK5) and glycogen synthase kinase 3 beta (GSK3B) in human microglia cells. These drugs were discovered using different data sets. Sildenafil was found using a high-quality literature-based AD endophenotype module (available as AlzGPS data set “V1 AD-seed”) containing 144 genes. Pioglitazone was found using 103 high-confidence AD risk genes (available as AlzGPS data set “V4 AD-inferred-GWAS-risk-genes”) identified by GWAS [13].

**Fig. 4:**
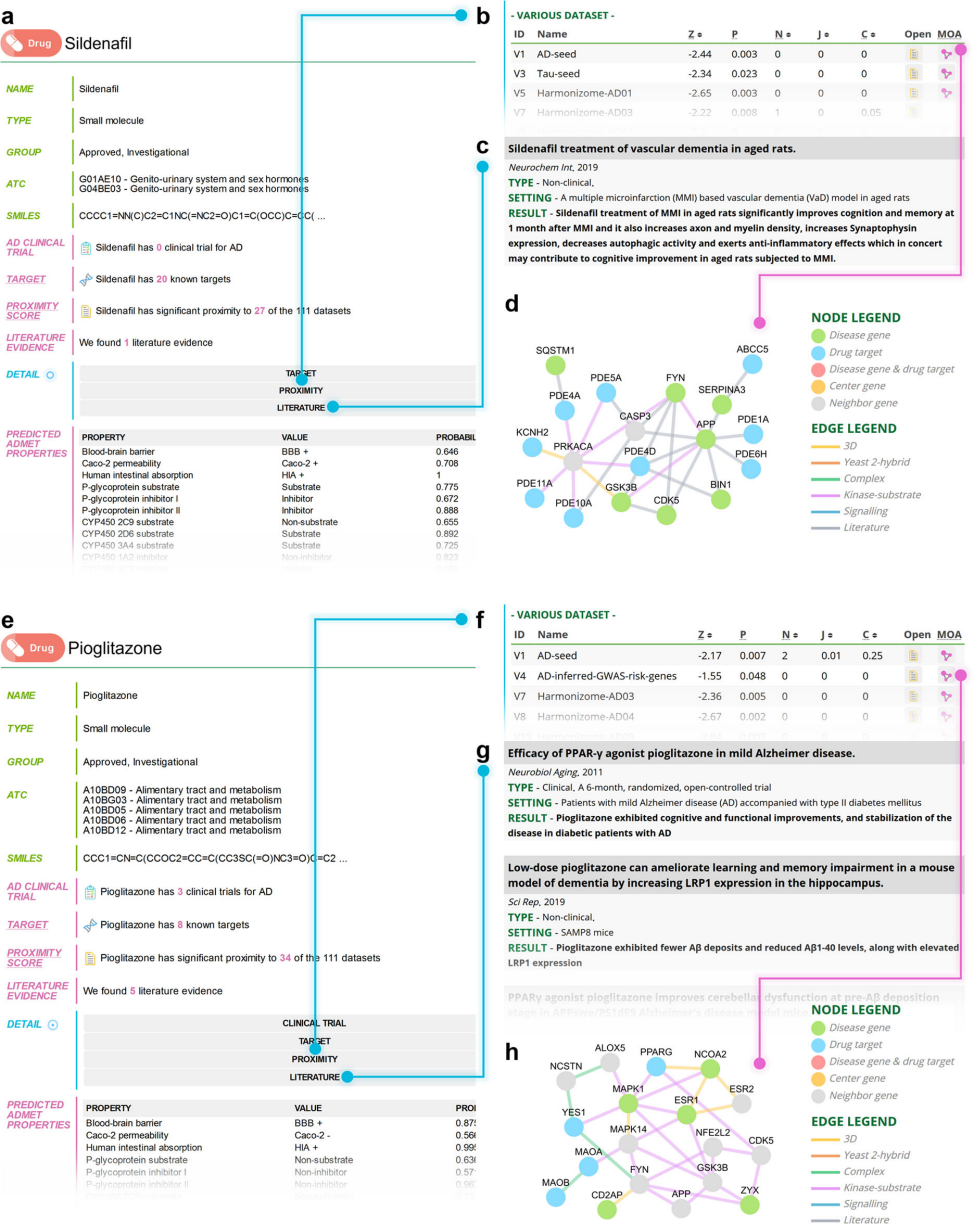
Case study—drug repurposing. Sildenafil and pioglitazone are used as examples to demonstrate how to use AlzGPS for drug repurposing. **a** Basic information for sildenafil. **b** Network proximity results for sildenafil. **c** Literature evidence for sildenafil. **d** Inferred mechanism-of-action for sildenafil targeting the “V1 AD-seed” data set, which contains 144 high-quality literature-based Alzheimer’s disease (AD) endophenotype genes. **e** Basic information for pioglitazone. **f** Network proximity results for pioglitazone. **g** Five studies were found that were related to treating AD with pioglitazone. **h** Inferred mechanism-of-action for pioglitazone targeting the “V4 AD-inferred-GWAS-risk-genes” data set which contains 103 high-confidence AD risk genes identified using genome-wide association studies

AlzGPS provides a list-view of the network proximity results of all the drugs organized by their first-level ATC code, which can be found in the “DRUG CLASS” tab (Fig. 2b). The drugs are ranked by the number of significant proximities to the data sets. Sildenafil is in the top four of the 148 drugs under the ATC code G “Genito-urinary system and sex hormones” with network proximity results, the top three being vardenafil, ibuprofen, and gentian violet cation. Pioglitazone is in the top six of the 226 drugs under the ATC code A “Alimentary tract and metabolism,” following tetracycline, human insulin, epinephrine, cholecalciferol, and teduglutide. Both drugs achieved high numbers of significant proximities to the expression data set. Next, we examined the basic information of these drugs (Fig. 4a, e). Both drugs are predicted to be BBB penetrable. Sildenafil has 20 known targets and is significantly proximal to 27 of the 111 data sets (Fig. 4a). We found one non-clinical study that reported that sildenafil treatment improves cognition and memory of vascular dementia in aged rats [43] (Fig. 4c). As noted, we identified the potential of sildenafil against AD using the AD endophenotype module (Fig. 4b, *Z* = − 2.44, *P* = 0.003). Then, clicking the corresponding “MOA (mechanism-of-action)” button opened the inferred MOA network for sildenafil and the data set (Fig. 4d). Although sildenafil does not target the genes in the data set (green) directly, it can potentially alter them through PPIs with its targets (blue).

Pioglitazone has 8 known targets and is significantly proximal to 34 data sets (Fig. 4e). Five studies containing both clinical and non-clinical data were found to be related to treating AD with pioglitazone. For example, a clinical study showed that pioglitazone can improve cognition in AD patients with type II diabetes [44] (Fig. 4g). Similarly, network results and associated MOA networks suggested that pioglitazone can affect AD risk genes through PPIs (Fig. 4f, h).

### Validation studies

Once candidate agents are identified on AlzGPS, a variety of validation steps can be pursued [6]. The agent can be tested in animal model systems of AD pathology to evaluate the predicted MOA of behavioral and biological effects. Since these are repurposed agents and have been used for other indications in human healthcare, electronic medical records can be interrogated to determine if there are notable effects on AD incidence, prevalence, or rate of progression. Both these methods are imperfect since animal models have rarely been predictive of human response, and doses and duration of exposures may be different for indications other than AD in which the candidate agents are used. The ultimate assessment that could make an agent available for human care is success in a clinical trial and nominated agents must eventually be submitted to trials. If repurposed agents are not entered into trials because of intellectual property limitations or other challenges, the information from AlzGPS may be useful in identifying druggable disease pathways or providing seed structures that provide a basis for creation of related novel agents with similar MOAs.

## Discussion

Dr. Alois Alzheimer first described the condition in 1907, but scientists have not been able to develop any disease-modifying treatments for AD since. In this study, we developed a computational platform, termed AlzGPS (https://alzgps.lerner.ccf.org), which will advance genome-informed Alzheimer’s patient care and therapeutic development, by leveraging all existing multi-omics knowledge and data. To be specific, AlzGPS enables searching, sharing, visualizing, querying, and analyzing multi-omics (genomics, transcriptomics, proteomics, metabolomics, and interactomics), different types of heterogeneous bio-networks, and clinical databases for genome-informed target identification and drug repurposing for potential treatment of AD. In addition, drug candidates prioritized by AlzGPS may offer possible tool compounds for investigation of disease biology or pathobiology of AD. We believe that AlzGPS will be a valuable tool for the AD drug discovery community by providing (1) (manually curated) abundant diverse information of AD multi-omics data sets, genes, and drugs; (2) drug repurposing results using state-of-the-art network proximity approaches for novel insights; and (3) highly interactive and intuitive web interface with informative network visualizations. To the best knowledge of the authors, this study presents the first AD multi-omics framework using both network-based methodologies and genome-informed precision medicine drug discovery for AD.

We acknowledge several potential limitations in current AlzGPS. First, we assembled multi-omics data and clinical trial data from diverse sources. Data harmonization is a crucial issue which should be addressed in the future, possibly through machine learning approaches. Second, although we assembled comprehensive PPIs based on our sizeable efforts, incompleteness of human protein-protein interactome data and potential literature bias may influence performance of AlzGPS. For example, well-studied genes (such as *APOE*, *MAPT*, and *BACE1*) are top prioritized target candidates as they have more accumulating PPIs, genetic and genomic information. In addition, the current implementation of AlzGPS does not differentiate the allele-specific expression. We will integrate more isoform-specific expression profiles (such as *APOE* ε2/ε3/ε4) in the AlzGPS in the future. Although we integrated large-scale genetic data from meta-analyses of GWAS, whole-genome/exome sequencing data for AD are missing in current AlzGPS. We will integrate high-throughput next-generation DNA sequencing from multiple national AD genome projects, including the Alzheimer’s Disease Sequencing Project (ADSP) and the Alzheimer’s Disease Neuroimaging Initiative (ADNI), especially DNA/RNA sequencing from minority population, which will provide unbiased genomic resources to prioritize novel drug targets. Systematic evaluation of pharmacokinetic properties (including brain penetration) for drugs using in silico approaches and publicly available in vitro and in vivo assays is highly encouraged in the future. Finally, we will integrate clinical trial and approved drug information from other sources, including European Medicines Agency, Pharmaceuticals and Medical Devices Agency at Japan, and the China Food and Drug Administration, to advance the international Alzheimer’s research communities under the AlzGPS framework. We will continue to add more types of omics data and update AlzGPS annually or when a large amount of new data is available.

## Conclusions

In summary, AlzGPS presents the first comprehensive in silico tool for human genome-informed precision medicine drug discovery for AD. AlzGPS contains rich and diverse information connecting genetics, genomics, proteomics, and metabolomics for disease pathobiology, and drugs for AD target identification and drug repurposing. It utilizes multiple biological networks and omics data, and provides network-based drug repurposing results with network visualizations. From a translational perspective, if broadly applied, AlzGPS will offer a powerful tool for prioritizing biologically relevant targets and clinically relevant repurposed drug candidates and tool compounds for multi-omics-informed discovery in AD and other neurodegenerative diseases.

## Supplementary Information

**Additional file 1: Table S1.** All data sets in AlzGPS.

## Data Availability

All the data in AlzGPS can be freely accessed without registration requirement at https://alzgps.lerner.ccf.org.
