# Supplementary material for: AlzGPS: a genome-wide positioning systems platform to catalyze multi-omics for Alzheimer’s drug discovery
Source: Alzheimers Res Ther. 2021 Jan 13;13:24. doi: 10.1186/s13195-020-00760-w (PMC7804907; doi:10.1186/s13195-020-00760-w)
Supplement: Supplementary file 1 — Additional file 1: Table S1. All data sets in AlzGPS. [file 13195_2020_760_MOESM1_ESM.pdf]

Table S1. All data sets in AlzGPS.

Dataset ID: E1
Taxon: Human
Omic: Transcriptome
Method: Microarray
Region: Brain
Group (sample): 30 EAD vs. 173 controls
Criteria: FDR < 0.01, |FC| > 1.2
GEO: GSE48350
PMID: 23273601
Note: EAD, Braak III or IV
Source: GEO2R

Dataset ID: E2
Taxon: Human
Omic: Transcriptome
Method: Microarray
Region: Brain
Group (sample): 180 EAD vs. 214 controls
Criteria: FDR < 0.01, |FC| > 1.2
GEO: GSE84422
PMID: 27799057
Note: EAD, Probable AD (Braak III or IV)
Source: GEO2R

Dataset ID: E3
Taxon: Human
Omic: Transcriptome
Method: Microarray
Region: Brain
Group (sample): 180 EAD vs. 214 controls
Criteria: FDR < 0.01, |FC| > 1.2
GEO: GSE84422
PMID: 27799057
Note: EAD, Probable AD (Braak III or IV)
Source: GEO2R

Dataset ID: E4
Taxon: Human
Omic: Transcriptome
Method: Microarray
Region: Brain
Group (sample): 6 EAD vs. 8 controls
Criteria: p<0.01 (Paper)
GEO: GSE12685

PMID: 19295912
Note: controls (MMSE 30-25) and "Incipient AD" (MMSE 21-26, with MCI 24-26 and mild AD 21-23)
Source: Table S2

Dataset ID: E5
Taxon: Human
Omic: Transcriptome
Method: Microarray
Region: Hippocampus
Group (sample): 31 LAD vs. 32 controls
Criteria: FDR < 0.01, |FC| > 1.2
GEO: GSE29378
PMID: 23705665
Note: LAD, Braak V or VI
Source: GEO2R

Dataset ID: E6
Taxon: Human
Omic: Transcriptome
Method: Microarray
Region: Brain
Group (sample): 42 LAD vs. 173 controls
Criteria: FDR < 0.01, |FC| > 1.2
GEO: GSE48350
PMID: 23273601
Note: LAD, Braak V or VI
Source: GEO2R

Dataset ID: E7
Taxon: Human
Omic: Transcriptome
Method: Microarray
Region: Brain
Group (sample): 328 LAD vs. 214 controls
Criteria: FDR < 0.01, |FC| > 1.2
GEO: GSE84422
PMID: 27799057
Note: LAD, Definite AD (Braak V or VI)
Source: GEO2R

Dataset ID: E8
Taxon: Human
Omic: Transcriptome
Method: Microarray
Region: Brain

Group (sample): 328 LAD vs. 214 controls
Criteria: FDR < 0.01, |FC| > 1.2
GEO: GSE84422
PMID: 27799057
Note: LAD, Definite AD (Braak V or VI)
Source: GEO2R

Dataset ID: E9
Taxon: Human
Omic: Transcriptome
Method: Bulk RNA-seq
Region: Hippocampus
Group (sample): 4 LAD vs. 4 controls
Criteria: PFP<0.1 (Paper)
GEO: GSE67333
PMID: 26402107
Note: LAD, Braak V or VI
Source: File S2

Dataset ID: E10
Taxon: Human
Omic: Transcriptome
Method: Bulk RNA-seq
Region: Hippocampus
Group (sample): 6 LAD vs. 6 controls
Criteria: |log2(FC)|> 1 and corrected P value < 0.05 (Paper)
PMID: 29523845
Note: LAD, Braak V or VI
Source: Table S2

Dataset ID: E11
Taxon: Human
Omic: Transcriptome
Method: Bulk RNA-seq
Region: Hippocampus
Group (sample): 20 LAD vs. 10 controls
Criteria: DE score >0.1
PMID: 30497016
Note: LAD, Braak V or VI
Source: Table S1

Dataset ID: E12
Taxon: Human
Omic: Transcriptome
Method: Single Cell
Region: Brain

Cell type: Excitatory neurons (Ex)
Group (sample): 24 AD vs. 24 controls
Criteria: FDR < 0.01, |FC| > 1.2, Poisson mixed-model FDR < 0.05
PMID: 31042697
Note: A spectrum of mild to severe Aβ and other pathologies (AD-pathology)
Source: Table S2

Dataset ID: E13
Taxon: Human
Omic: Transcriptome
Method: Single Cell
Region: Brain
Cell type: Inhibitory neurons (In)
Group (sample): 24 AD vs. 24 controls
Criteria: FDR < 0.01, |FC| > 1.2, Poisson mixed-model FDR < 0.05
PMID: 31042697
Note: A spectrum of mild to severe Aβ and other pathologies (AD-pathology)
Source: Table S2

Dataset ID: E14
Taxon: Human
Omic: Transcriptome
Method: Single Cell
Region: Brain
Cell type: Astrocytes (Ast)
Group (sample): 24 AD vs. 24 controls
Criteria: FDR < 0.01, |FC| > 1.2, Poisson mixed-model FDR < 0.05
PMID: 31042697
Note: A spectrum of mild to severe Aβ and other pathologies (AD-pathology)
Source: Table S2

Dataset ID: E15
Taxon: Human
Omic: Transcriptome
Method: Single Cell
Region: Brain
Cell type: Oligodendrocytes (Oli)
Group (sample): 24 AD vs. 24 controls
Criteria: FDR < 0.01, |FC| > 1.2, Poisson mixed-model FDR < 0.05
PMID: 31042697
Note: A spectrum of mild to severe Aβ and other pathologies (AD-pathology)
Source: Table S2

Dataset ID: E16
Taxon: Human
Omic: Transcriptome

Method: Single Cell
Region: Brain
Cell type: Microglia (Mic)
Group (sample): 24 AD vs. 24 controls
Criteria: FDR < 0.01, |FC| > 1.2, Poisson mixed-model FDR < 0.05
PMID: 31042697
Note: A spectrum of mild to severe Aβ and other pathologies (AD-pathology)
Source: Table S2

Dataset ID: E17
Taxon: Human
Omic: Transcriptome
Method: Single Cell
Region: Brain
Cell type: Oligodendrocyte precursor cells (OPC)
Group (sample): 24 AD vs. 24 controls
Criteria: FDR < 0.01, |FC| > 1.2, Poisson mixed-model FDR < 0.05
PMID: 31042697
Note: A spectrum of mild to severe Aβ and other pathologies (AD-pathology)
Source: Table S2

Dataset ID: E18
Taxon: Human
Omic: Transcriptome
Method: Single Cell
Region: Brain
Cell type: Excitatory neurons (Ex)
Group (sample): EAD vs. controls
Criteria: FDR < 0.01, |FC| > 1.2, Poisson mixed-model FDR < 0.05
PMID: 31042697
Note: EAD, amyloid burden, but modest neurofibrillary tangles and cognitive impairment
Source: Table S2

Dataset ID: E19
Taxon: Human
Omic: Transcriptome
Method: Single Cell
Region: Brain
Cell type: Inhibitory neurons (In)
Group (sample): EAD vs. controls
Criteria: FDR < 0.01, |FC| > 1.2, Poisson mixed-model FDR < 0.05
PMID: 31042697
Note: EAD, amyloid burden, but modest neurofibrillary tangles and cognitive impairment
Source: Table S2

Dataset ID: E20

Taxon: Human
Omic: Transcriptome
Method: Single Cell
Region: Brain
Cell type: Astrocytes (Ast)
Group (sample): EAD vs. controls
Criteria: FDR < 0.01, |FC| > 1.2, Poisson mixed-model FDR < 0.05
PMID: 31042697
Note: EAD, amyloid burden, but modest neurofibrillary tangles and cognitive impairment
Source: Table S2

Dataset ID: E21
Taxon: Human
Omic: Transcriptome
Method: Single Cell
Region: Brain
Cell type: Oligodendrocytes (Oli)
Group (sample): EAD vs. controls
Criteria: FDR < 0.01, |FC| > 1.2, Poisson mixed-model FDR < 0.05
PMID: 31042697
Note: EAD, amyloid burden, but modest neurofibrillary tangles and cognitive impairment
Source: Table S2

Dataset ID: E22
Taxon: Human
Omic: Transcriptome
Method: Single Cell
Region: Brain
Cell type: Microglia (Mic)
Group (sample): EAD vs. controls
Criteria: FDR < 0.01, |FC| > 1.2, Poisson mixed-model FDR < 0.05
PMID: 31042697
Note: EAD, amyloid burden, but modest neurofibrillary tangles and cognitive impairment
Source: Table S2

Dataset ID: E23
Taxon: Human
Omic: Transcriptome
Method: Single Cell
Region: Brain
Cell type: Oligodendrocyte precursor cells (OPC)
Group (sample): EAD vs. controls
Criteria: FDR < 0.01, |FC| > 1.2, Poisson mixed-model FDR < 0.05
PMID: 31042697
Note: EAD, amyloid burden, but modest neurofibrillary tangles and cognitive impairment
Source: Table S2

Dataset ID: E24
Taxon: Human
Omic: Transcriptome
Method: Single Cell
Region: Brain
Cell type: Excitatory neurons (Ex)
Group (sample): LAD vs. EAD
Criteria: FDR < 0.01, |FC| > 1.2, Poisson mixed-model FDR < 0.05
PMID: 31042697
Note: LAD, higher amyloid, and also elevated neurofibrillary tangles, global pathology, and cognitive impairment
Source: Table S2

Dataset ID: E25
Taxon: Human
Omic: Transcriptome
Method: Single Cell
Region: Brain
Cell type: Inhibitory neurons (In)
Group (sample): LAD vs. EAD
Criteria: FDR < 0.01, |FC| > 1.2, Poisson mixed-model FDR < 0.05
PMID: 31042697
Note: LAD, higher amyloid, and also elevated neurofibrillary tangles, global pathology, and cognitive impairment
Source: Table S2

Dataset ID: E26
Taxon: Human
Omic: Transcriptome
Method: Single Cell
Region: Brain
Cell type: Astrocytes (Ast)
Group (sample): LAD vs. EAD
Criteria: FDR < 0.01, |FC| > 1.2, Poisson mixed-model FDR < 0.05
PMID: 31042697
Note: LAD, higher amyloid, and also elevated neurofibrillary tangles, global pathology, and cognitive impairment
Source: Table S2

Dataset ID: E27
Taxon: Human
Omic: Transcriptome
Method: Single Cell
Region: Brain
Cell type: Oligodendrocytes (Oli)

Group (sample): LAD vs. EAD
Criteria: FDR < 0.01, |FC| > 1.2, Poisson mixed-model FDR < 0.05
PMID: 31042697
Note: LAD, higher amyloid, and also elevated neurofibrillary tangles, global pathology, and cognitive impairment
Source: Table S2

Dataset ID: E28
Taxon: Human
Omic: Transcriptome
Method: Single Cell
Region: Brain
Cell type: Microglia (Mic)
Group (sample): LAD vs. EAD
Criteria: FDR < 0.01, |FC| > 1.2, Poisson mixed-model FDR < 0.05
PMID: 31042697
Note: LAD, higher amyloid, and also elevated neurofibrillary tangles, global pathology, and cognitive impairment
Source: Table S2

Dataset ID: E29
Taxon: Human
Omic: Transcriptome
Method: Single Cell
Region: Brain
Cell type: Oligodendrocyte precursor cells (OPC)
Group (sample): LAD vs. EAD
Criteria: FDR < 0.01, |FC| > 1.2, Poisson mixed-model FDR < 0.05
PMID: 31042697
Note: LAD, higher amyloid, and also elevated neurofibrillary tangles, global pathology, and cognitive impairment
Source: Table S2

Dataset ID: E30
Taxon: Human
Omic: Transcriptome
Method: Microarray
Region: Human fibroblasts cell
Cell type: Neural progenitor cells
Group (sample): 5 sporadic AD vs. 5 controls
Criteria: FDR < 0.05, |FC| > 1.5
GEO: GSE117589
PMID: 30699343
Note: Sporadic AD
Source: Table S3

Dataset ID: E31
Taxon: Human
Omic: Transcriptome
Method: Microarray
Region: Human fibroblasts cell
Cell type: Neural cells
Group (sample): 6 sporadic AD vs. 5 controls
Criteria: FDR < 0.05, |FC| > 1.5
GEO: GSE117589
PMID: 30699343
Note: Sporadic AD
Source: Table S4

Dataset ID: E32
Taxon: Mouse
Omic: Transcriptome
Method: Microarray
Genetic BG: C57Bl/6J
Model: HO-TASTPM
Age: 4 months
Region: Hippocampus
Group (sample): 4 AD mice vs. 9 controls
Criteria: FDR < 0.05, |FC| > 1.5
GEO: GSE64398
PMID: 25620700
Note: Mouse genes converted to human orthologs
Source: GEO2R

Dataset ID: E33
Taxon: Mouse
Omic: Transcriptome
Method: Microarray
Genetic BG: C57Bl/6J
Model: HO-TASTPM
Age: 8 months
Region: Hippocampus
Group (sample): 4 AD mice vs. 9 controls
Criteria: FDR < 0.05, |FC| > 1.5
GEO: GSE64398
PMID: 25620700
Note: Mouse genes converted to human orthologs
Source: GEO2R

Dataset ID: E34
Taxon: Mouse
Omic: Transcriptome

Method: Microarray
Genetic BG: C57Bl/6J
Model: HO-TASTPM
Age: 18 months
Region: Hippocampus
Group (sample): 4 AD mice vs. 9 controls
Criteria: FDR < 0.05, |FC| > 1.5
GEO: GSE64398
PMID: 25620700
Note: Mouse genes converted to human orthologs
Source: GEO2R

Dataset ID: E35
Taxon: Mouse
Omic: Transcriptome
Method: Microarray
Genetic BG: C57Bl/6J
Model: HO-TASTPM
Age: 4 months
Region: Frontal cortex
Group (sample): 4 AD mice vs. 9 controls
Criteria: FDR < 0.05, |FC| > 1.5
GEO: GSE64398
PMID: 25620700
Note: Mouse genes converted to human orthologs
Source: GEO2R

Dataset ID: E36
Taxon: Mouse
Omic: Transcriptome
Method: Microarray
Genetic BG: C57Bl/6J
Model: HO-TASTPM
Age: 8 months
Region: Frontal cortex
Group (sample): 4 AD mice vs. 9 controls
Criteria: FDR < 0.05, |FC| > 1.5
GEO: GSE64398
PMID: 25620700
Note: Mouse genes converted to human orthologs
Source: GEO2R

Dataset ID: E37
Taxon: Mouse
Omic: Transcriptome
Method: Microarray

Genetic BG: C57Bl/6J
Model: HO-TASTPM
Age: 18 months
Region: Frontal cortex
Group (sample): 3 AD mice vs. 7 controls
Criteria: FDR < 0.05, |FC| > 1.5
GEO: GSE64398
PMID: 25620700
Note: Mouse genes converted to human orthologs
Source: GEO2R

Dataset ID: E38
Taxon: Mouse
Omic: Transcriptome
Method: Microarray
Genetic BG: C57Bl/6J
Model: APP/PS1
Age: 8 months
Region: Brain
Cell type: Microglia (Mic)
Group (sample): 5 AD mice vs. 5 controls
Criteria: FDR < 0.05, |FC| > 1.5
GEO: GSE65067
PMID: 25728668
Note: Mouse genes converted to human orthologs
Source: GEO2R

Dataset ID: E39
Taxon: Mouse
Omic: Transcriptome
Method: Microarray
Genetic BG: C57Bl/6J
Model: APP/PS1
Age: 5 months
Region: Frontal cortex
Group (sample): 9 AD mice vs. 12 controls
Criteria: FDR < 0.05, |FC| > 1.5
GEO: GSE74438
Note: Mouse genes converted to human orthologs
Source: GEO2R

Dataset ID: E40
Taxon: Mouse
Omic: Transcriptome
Method: Microarray
Genetic BG: C57Bl/6J

Model: APP/PS1
Age: 5 months
Region: Hippocampus
Group (sample): 9 AD mice vs. 12 controls
Criteria: FDR < 0.05, |FC| > 1.5
GEO: GSE74438
Note: Mouse genes converted to human orthologs
Source: GEO2R

Dataset ID: E41
Taxon: Mouse
Omic: Transcriptome
Method: Microarray
Genetic BG: C57Bl/6J
Model: APP/PS1
Age: 5 months
Region: Hippocampus
Group (sample): 8 AD mice vs. 11 controls
Criteria: FDR < 0.05, |FC| > 1.5
GEO: GSE74437
Note: Mouse genes converted to human orthologs
Source: GEO2R

Dataset ID: E42
Taxon: Mouse
Omic: Transcriptome
Method: Microarray
Genetic BG: C57Bl/6J
Model: APP/PS1
Age: 5 months
Region: Frontal cortex
Group (sample): 8 AD mice vs. 11 controls
Criteria: FDR < 0.05, |FC| > 1.5
GEO: GSE74437
Note: Mouse genes converted to human orthologs
Source: GEO2R

Dataset ID: E43
Taxon: Mouse
Omic: Transcriptome
Method: Microarray
Genetic BG: A cross between C57BL/6J and C3H/HeJ
Model: APP/PS1
Age: 15-18 months
Region: Brain
Cell type: Microglia (Mic)

Group (sample): 7 AD mice vs. 7 controls
Criteria: FDR < 0.05, |FC| > 1.5
GEO: GSE74615
PMID: 25002035
Note: Mouse genes converted to human orthologs
Source: GEO2R

Dataset ID: E44
Taxon: Mouse
Omic: Transcriptome
Method: Microarray
Genetic BG: A cross between FVB/N(TRE-Tau) and 129S6(CaMKIIα-tTA)
Model: rTg4510
Age: 4 months
Region: Hippocampus
Group (sample): 4 AD mice vs. 4 controls
Criteria: FDR < 0.05, |FC| > 1.5
GEO: GSE53480
PMID: 25069841
Note: Mouse genes converted to human orthologs
Source: GEO2R

Dataset ID: E45
Taxon: Mouse
Omic: Transcriptome
Method: Microarray
Genetic BG: A cross between FVB/N(TRE-Tau) and 129S6(CaMKIIα-tTA)
Model: rTg4510
Age: 4-6 months
Region: Hippocampus
Group (sample): 16 AD mice vs. 20 controls
Criteria: FDR < 0.05, |FC| > 1.5
GEO: GSE56772
Note: Mouse genes converted to human orthologs
Source: GEO2R

Dataset ID: E46
Taxon: Mouse
Omic: Transcriptome
Method: Microarray
Genetic BG: A cross between FVB/N(TRE-Tau) and 129S6(CaMKIIα-tTA)
Model: rTg4510
Age: 6 months
Region: Hippocampus
Group (sample): 17 AD mice vs. 17 controls
Criteria: FDR < 0.05, |FC| > 1.5

GEO: GSE57583
Note: Mouse genes converted to human orthologs
Source: GEO2R

Dataset ID: E47
Taxon: Mouse
Omic: Transcriptome
Method: Microarray
Genetic BG: C57Bl/6J
Model: Tau P301L
Age: 18 months
Region: Hippocampus
Group (sample): 3 AD mice vs. 7 controls
Criteria: FDR < 0.05, |FC| > 1.5
GEO: GSE64398
PMID: 25620700
Note: Mouse genes converted to human orthologs
Source: GEO2R

Dataset ID: E48
Taxon: Mouse
Omic: Transcriptome
Method: Microarray
Genetic BG: C57Bl/6J
Model: Tau P301L
Age: 18 months
Region: Frontal cortex
Group (sample): 3 AD mice vs. 7 controls
Criteria: FDR < 0.05, |FC| > 1.5
GEO: GSE64398
PMID: 25620700
Note: Mouse genes converted to human orthologs
Source: GEO2R

Dataset ID: E49
Taxon: Mouse
Omic: Transcriptome
Method: Microarray
Genetic BG: C57Bl/6J
Model: Tau P301L
Age: 18 months
Region: Cerebellum
Group (sample): 3 AD mice vs. 7 controls
Criteria: FDR < 0.05, |FC| > 1.5
GEO: GSE64398
PMID: 25620700

Note: Mouse genes converted to human orthologs
Source: GEO2R

Dataset ID: E50
Taxon: Mouse
Omic: Transcriptome
Method: Bulk RNA-seq
Genetic BG: A cross between C57BL/6J females (B6) and C3H/HeJ males (C3)
Model: TgCRND8
Age: 1.5 months
Region: Cortex
Group (sample): 4 AD mice vs. 6 controls
Criteria: T-test, P < 0.01
PMID: 30189875
Note: Mouse genes converted to human orthologs
Source: Table S1

Dataset ID: E51
Taxon: Mouse
Omic: Transcriptome
Method: Bulk RNA-seq
Genetic BG: A cross between C57BL/6J females (B6) and C3H/HeJ males (C3)
Model: TgCRND8
Age: 3 months
Region: Cortex
Group (sample): 6 AD mice vs. 6 controls
Criteria: T-test, P < 0.01
PMID: 30189875
Note: Mouse genes converted to human orthologs
Source: Table S1

Dataset ID: E52
Taxon: Mouse
Omic: Transcriptome
Method: Bulk RNA-seq
Genetic BG: A cross between C57BL/6J females (B6) and C3H/HeJ males (C3)
Model: TgCRND8
Age: 4.5 months
Region: Cortex
Group (sample): 3 AD mice vs. 4 controls
Criteria: T-test, P < 0.01
PMID: 30189875
Note: Mouse genes converted to human orthologs
Source: Table S1

Dataset ID: E53

Taxon: Mouse
Omic: Transcriptome
Method: Bulk RNA-seq
Genetic BG: A cross between C57BL/6Boy mice to SJL/J mice
Model: Tg2576
Age: 3 months
Region: Cortex
Group (sample): 4 AD mice vs. 4 controls
Criteria: T-test, P < 0.01
PMID: 30189875
Note: Mouse genes converted to human orthologs
Source: Table S1

Dataset ID: E54
Taxon: Mouse
Omic: Transcriptome
Method: Bulk RNA-seq
Genetic BG: C57Bl/6J
Model: 5XFAD
Age: 3-6 months
Region: Brain
Group (sample): Young WT vs. young 5XFAD
Criteria: FDR < 0.05, |FC| > 1.5
PMID: 24795628
Note: Mouse genes converted to human orthologs
Source: Table 2

Dataset ID: E55
Taxon: Mouse
Omic: Transcriptome
Method: Bulk RNA-seq
Genetic BG: C57Bl/6J
Model: APP_PS1KI
Age: 6 months
Region: Brain
Group (sample): 5 AD mice vs. 5 controls
Criteria: FDR < 0.05
PMID: 26639971
Note: Mouse genes converted to human orthologs
Source: Table S2

Dataset ID: E56
Taxon: Mouse
Omic: Transcriptome
Method: Bulk RNA-seq
Genetic BG: A cross between C57BL/6J females (B6) and C3H/HeJ males (C3)

Model: TgCRND8
Age: 6 months
Region: Cortex
Group (sample): 5 AD mice vs. 5 controls
Criteria: T-test, P < 0.01
PMID: 30189875
Note: Mouse genes converted to human orthologs
Source: Table S1

Dataset ID: E57
Taxon: Mouse
Omic: Transcriptome
Method: Bulk RNA-seq
Genetic BG: A cross between C57BL/6Boy mice to SJL/J mice
Model: Tg2576
Age: 6 months
Region: Cortex
Group (sample): 4 AD mice vs. 4 controls
Criteria: T-test, P < 0.01
PMID: 30189875
Note: Mouse genes converted to human orthologs
Source: Table S1

Dataset ID: E58
Taxon: Mouse
Omic: Transcriptome
Method: Bulk RNA-seq
Genetic BG: C57Bl/6J
Model: APP/PSEN1
Age: 8 months
Region: Prefrontal cortex
Group (sample): 4 AD mice vs. 4 controls
Criteria: FDR < 0.05, |FC| > 1.2
PMID: 30283032
Note: Mouse genes converted to human orthologs
Source: Table S1

Dataset ID: E59
Taxon: Mouse
Omic: Transcriptome
Method: Bulk RNA-seq
Genetic BG: C57Bl/6J
Model: 5xFAD
Age: 6 months
Region: Brain
Cell type: Microglia (Mic)

Group (sample): Amyloid plaque-containing (XO4+) vs non-containing (XO4-) microglia
Criteria: FDR < 0.05, |FC| > 1.2
Note: Mouse genes converted to human orthologs
Source: doi.org/10.1101/639054

Dataset ID: E60
Taxon: Mouse
Omic: Transcriptome
Method: Bulk RNA-seq
Genetic BG: A cross between C57BL/6Boy mice to SJL/J mice
Model: Tg2576
Age: 9 months
Region: Cortex
Group (sample): 4 AD mice vs. 4 controls
Criteria: T-test, P < 0.01
PMID: 30189875
Note: Mouse genes converted to human orthologs
Source: Table S1

Dataset ID: E61
Taxon: Mouse
Omic: Transcriptome
Method: Bulk RNA-seq
Genetic BG: A cross between C57BL/6J females (B6) and C3H/HeJ males (C3)
Model: TgCRND8
Age: 10 months
Region: Cortex
Group (sample): 7 AD mice vs. 6 controls
Criteria: T-test, P < 0.01
PMID: 30189875
Note: Mouse genes converted to human orthologs
Source: Table S1

Dataset ID: E62
Taxon: Mouse
Omic: Transcriptome
Method: Bulk RNA-seq
Genetic BG: A cross between C57BL/6Boy mice to SJL/J mice
Model: Tg2576
Age: 12 months
Region: Cortex
Group (sample): 4 AD mice vs. 4 controls
Criteria: T-test, P < 0.01
PMID: 30189875
Note: Mouse genes converted to human orthologs
Source: Table S1

Dataset ID: E63
Taxon: Mouse
Omic: Transcriptome
Method: Bulk RNA-seq
Genetic BG: A cross between C57BL/6Boy mice to SJL/J mice
Model: Tg2576
Age: 15 months
Region: Cortex
Group (sample): 4 AD mice vs. 4 controls
Criteria: T-test, $P < 0.01$
PMID: 30189875
Note: Mouse genes converted to human orthologs
Source: Table S1

Dataset ID: E64
Taxon: Mouse
Omic: Transcriptome
Method: Bulk RNA-seq
Genetic BG: C57Bl/6J
Model: 5XFAD
Age: 12 months
Region: Brain
Group (sample): Aged WT vs. aged 5XFAD
Criteria: FDR < 0.05, |FC| > 1.5
PMID: 24795628
Note: Mouse genes converted to human orthologs
Source: Table 5

Dataset ID: E65
Taxon: Mouse
Omic: Transcriptome
Method: Bulk RNA-seq
Genetic BG: C57Bl/6J
Model: Tg4_42
Age: 12 months
Region: Brain
Group (sample): Aged WT vs. aged Tg4–42
Criteria: FDR < 0.05, |FC| > 1.5
PMID: 24795628
Note: Mouse genes converted to human orthologs
Source: Table 3

Dataset ID: E66
Taxon: Mouse
Omic: Transcriptome

Method: Bulk RNA-seq
Genetic BG: A cross between FVB/N(TRE-Tau) and 129S6(CaMKIIα-tTA)
Model: rTg4510
Age: 2 months
Region: Brain
Cell type: Microglia (Mic)
Group (sample): 4 AD mice vs. 4 controls
Criteria: FDR<0.05 and |FC|>1.5
GEO: GSE123467
PMID: 30558641
Note: Mouse genes converted to human orthologs
Source: Table S1

Dataset ID: E67
Taxon: Mouse
Omic: Transcriptome
Method: Bulk RNA-seq
Genetic BG: A cross between FVB/N(TRE-Tau) and 129S6(CaMKIIα-tTA)
Model: rTg4510
Age: 4 months
Region: Brain
Cell type: Microglia (Mic)
Group (sample): 3 AD mice vs. 3 controls
Criteria: FDR<0.05 and |FC|>1.5
GEO: GSE123467
PMID: 30558641
Note: Mouse genes converted to human orthologs
Source: Table S1

Dataset ID: E68
Taxon: Mouse
Omic: Transcriptome
Method: Bulk RNA-seq
Genetic BG: A cross between FVB/N(TRE-Tau) and 129S6(CaMKIIα-tTA)
Model: rTg4510
Age: 6 months
Region: Brain
Cell type: Microglia (Mic)
Group (sample): 4 AD mice vs. 4 controls
Criteria: FDR<0.05 and |FC|>1.5
GEO: GSE123467
PMID: 30558641
Note: Mouse genes converted to human orthologs
Source: Table S1

Dataset ID: E69

Taxon: Mouse
Omic: Transcriptome
Method: Bulk RNA-seq
Genetic BG: A cross between FVB/N(TRE-Tau) and 129S6(CaMKIIα-tTA)
Model: rTg4510
Age: 8 months
Region: Brain
Cell type: Microglia (Mic)
Group (sample): 3 AD mice vs. 3 controls
Criteria: FDR<0.05 and |FC|>1.5
GEO: GSE123467
PMID: 30558641
Note: Mouse genes converted to human orthologs
Source: Table S1

Dataset ID: E70
Taxon: Mouse
Omic: Proteome
Method: 10-plex tandem mass tag
Genetic BG: Crossing the 5XFAD strain with JNPL3 tau animals
Model: ADLPAPT
Age: 4 months
Region: Hippocampus
Group (sample): 3 AD mice vs. 3 controls
Criteria: p-value<0.05 for the Student's t-test; FDR<0.05 for the ANOVA test.
PMID: 29338754
Note: Mouse genes converted to human orthologs. Differentially expressed proteins represented by genes
Source: Table S3

Dataset ID: E71
Taxon: Mouse
Omic: Proteome
Method: 10-plex tandem mass tag
Genetic BG: Crossing the 5XFAD strain with JNPL3 tau animals
Model: ADLPAPT
Age: 7 months
Region: Hippocampus
Group (sample): 3 AD mice vs. 3 controls
Criteria: p-value<0.05 for the Student's t-test; FDR<0.05 for the ANOVA test.
PMID: 29338754
Note: Mouse genes converted to human orthologs. Differentially expressed proteins represented by genes
Source: Table S3

Dataset ID: E72

Taxon: Mouse
Omic: Proteome
Method: 10-plex tandem mass tag
Genetic BG: Crossing the 5XFAD strain with JNPL3 tau animals
Model: ADLPAPT
Age: 10 months
Region: Hippocampus
Group (sample): 3 AD mice vs. 3 controls
Criteria: p-value<0.05 for the Student's t-test; FDR<0.05 for the ANOVA test.
PMID: 29338754
Note: Mouse genes converted to human orthologs. Differentially expressed proteins represented by genes
Source: Table S3

Dataset ID: E73
Taxon: Mouse
Omic: Proteome
Method: 10-plex tandem mass tag
Genetic BG: C57BL6
Model: 5XFAD
Age: 4 months
Region: Hippocampus
Group (sample): 3 AD mice vs. 3 controls
Criteria: p-value<0.05 for the Student's t-test; FDR<0.05 for the ANOVA test.
PMID: 29338754
Note: Mouse genes converted to human orthologs. Differentially expressed proteins represented by genes
Source: Table S3

Dataset ID: E74
Taxon: Mouse
Omic: Proteome
Method: 10-plex tandem mass tag
Genetic BG: C57BL6
Model: 5XFAD
Age: 7 months
Region: Hippocampus
Group (sample): 3 AD mice vs. 3 controls
Criteria: p-value<0.05 for the Student's t-test; FDR<0.05 for the ANOVA test.
PMID: 29338754
Note: Mouse genes converted to human orthologs. Differentially expressed proteins represented by genes
Source: Table S3

Dataset ID: E75
Taxon: Mouse

Omic: Proteome
Method: 10-plex tandem mass tag
Genetic BG: C57BL6
Model: 5XFAD
Age: 10 months
Region: Hippocampus
Group (sample): 3 AD mice vs. 3 controls
Criteria: p-value<0.05 for the Student's t-test; FDR<0.05 for the ANOVA test.
PMID: 29338754
Note: Mouse genes converted to human orthologs. Differentially expressed proteins represented by genes
Source: Table S3

Dataset ID: E76
Taxon: Mouse
Omic: Proteome
Method: Tandem mass spectrometry
Genetic BG: C57BL6
Model: 5XFAD
Age: 3 months
Region: Brain
Group (sample): 5XFAD_3M vs controls_3M
Criteria: FDR < 0.05
PMID: 29186695
Note: Mouse genes converted to human orthologs. Differentially expressed proteins represented by genes
Source: Integrated DEPs from 5XFAD-3M-Hip, 5XFAD-3M-Cere, and 5XFAD-3M-FC. (Table S2)

Dataset ID: E77
Taxon: Mouse
Omic: Proteome
Method: Tandem mass spectrometry
Genetic BG: C57BL6
Model: 5XFAD
Age: 12 months
Region: Brain
Group (sample): 5XFAD_12M vs controls_12M
Criteria: FDR < 0.05
PMID: 29186695
Note: Mouse genes converted to human orthologs. Differentially expressed proteins represented by genes
Source: Integrated DEPs from 5XFAD-12M-Hip, 5XFAD-12M-Cere, and 5XFAD-12M-FC. (Table S2)

Dataset ID: E78

Taxon: Mouse
Omic: Proteome
Method: Tandem mass spectrometry
Genetic BG: C57BL6
Model: hAPP
Age: 3 months
Region: Brain
Group (sample): hAPP_3M vs controls_3M
Criteria: FDR < 0.05
PMID: 29186695
Note: Mouse genes converted to human orthologs. Differentially expressed proteins represented by genes
Source: Integrated DEPs from hAPP-3M-Hip, hAPP-3M-Cere, and hAPP-3M-FC. (Table S1)

Dataset ID: E79
Taxon: Mouse
Omic: Proteome
Method: Tandem mass spectrometry
Genetic BG: C57BL6
Model: hAPP
Age: 12 months
Region: Brain
Group (sample): hAPP_12M vs controls_12M
Criteria: FDR < 0.05
PMID: 29186695
Note: Mouse genes converted to human orthologs. Differentially expressed proteins represented by genes
Source: Integrated DEPs from hAPP-12M-Hip, hAPP-12M-Cere, and hAPP-12M-FC.(Table S1)

Dataset ID: E80
Taxon: Fruit fly
Omic: Transcriptome
Method: Microarray
Criteria: FDR < 0.05
GEO: GSE48681
PMID: 24336499
Note: Fruit fly genes converted to human orthologs
Source: Table S1, 233 (day-matched) and 636 (survival-matched) DEGs, with a total of 712 genes combined

Dataset ID: E81
Taxon: Fruit fly
Omic: Transcriptome
Method: Bulk RNA-seq

Group (sample): eGRL_Aß42 vs eGRL_Control
Criteria: FDR < 0.05, |FC| > 1.5
PMID: 29598827
Note: Fruit fly genes converted to human orthologs
Source: eGRL_Aß42 vs eGRL_Control, Table S1

Dataset ID: E82
Taxon: Fruit fly
Omic: Transcriptome
Method: Bulk RNA-seq
Group (sample): GGRL_Tau vs GGRL_Control
Criteria: FDR < 0.05, |FC| > 1.5
PMID: 29598827
Note: Fruit fly genes converted to human orthologs
Source: GGRL_Tau vs GGRL_Control, Table S1

Dataset ID: E83
Taxon: C. elegans
Omic: Transcriptome
Method: Bulk RNA-seq
Criteria: FDR < 0.05, |FC| > 2
PMID: 28982592
Note: C. elegans genes converted to human orthologs
Source: Significant differential expressed genes between N2 and UM0002 (Aβ1–42 + anti-aggregating tau), Table S3

Dataset ID: E84
Taxon: C. elegans
Omic: Transcriptome
Method: Bulk RNA-seq
Criteria: FDR < 0.05, |FC| > 2
PMID: 28982592
Note: C. elegans genes converted to human orthologs
Source: Significant differential expressed genes between N2 and UM0001(Aβ1–42 + pro-aggregating tau) , Tabel S3

Dataset ID: M1
Taxon: Human
Omic: Metabolomics
Method: UPLC-HRMS (HILIC)
Region: Brain
Group (sample): 19 CN vs 21 LAD
Criteria: p < 0.05
PMID: 26717242
Note: Semi quantification
Source: J Proteome Res. 2016 Feb 5;15(2):608-18.

Dataset ID: M2
Taxon: Human
Omic: Metabolomics
Method: LC-MS/MS (C18)
Region: Serum
Group (sample): 370 CN vs 305 AD
Criteria: $p < 0.05$
PMID: 30337153
Note: Absolute quantification
Source: Schizophr Res. 2019 Apr;206:428-435.

Dataset ID: M3
Taxon: Human
Omic: Metabolomics
Method: HILIC-MS, GC-MS
Region: Brain (middle frontal gyrus, inferior temporal gyrus, cerebellum)
Group (sample): 14 Cont vs 14 AD
Criteria: $p < 0.05$
PMID: 28323825
Note: Semi quantification
Source: PLoS Med. 2017 Mar 21;14(3):e1002266.

Dataset ID: M4
Taxon: Human
Omic: Metabolomics
Method: Biocrates AbsoluteIDQ p180
Region: Brain (inferior temporal gyrus)
Group (sample): 14 CN, 15 ASYMAD, 15 AD
Criteria: chose the top 20 ranked metabolites from the SVM and RF algorithms, $p < 0.05$
PMID: 29370177
Note: Absolute quantification
Source: PLoS Med. 2018 Jan 25;15(1):e1002482.

Dataset ID: M5
Taxon: Human
Omic: Metabolomics
Method: CE-MS
Region: Brain (middle frontal gyrus, inferior temporal gyrus)
Group (sample): 13 CN, 13 ASYMAD, 17 AD
Criteria: FDR < 0.05
PMID: 31978055
Note: Absolute quantification
Source: PLoS Med. 2020 Jan 24;17(1):e1003012.

Dataset ID: M6

Taxon: Human
Omic: Metabolomics
Method: GC-MS
Region: Brain
Group (sample): 15 CN vs 14 LAD
Criteria: $p < 0.05$
PMID: 27069549
Note: Absolute quantification
Source: Am J Transl Res. 2016 Jan 15;8(1):154-65.

Dataset ID: M7
Taxon: Human
Omic: Metabolomics
Method: LC-MS, NMR
Region: Plasma
Group (sample): 10 CN, 12 MCI, 13 AD
Criteria: $p < 0.05$
PMID: 24041970
Note: Absolute quantification
Source: Neurobiol Aging. 2014 Feb;35(2):271-8.

Dataset ID: M8
Taxon: Human
Omic: Metabolomics
Method: LC-MS
Region: Plasma
Group (sample): 53 NC, 18 pre, 18 pos, 35 aMCI/AD
Criteria: $p < 0.05$
PMID: 24608097
Note: Absolute quantification
Source: Nat Med. 2014 Apr;20(4):415-8.

Dataset ID: M9
Taxon: Human
Omic: Metabolomics
Method: LC-TOF
Region: Brain (frontal, parietal)
Group (sample): 10 CN, vs 10 AD
Criteria: $p < 0.05$
PMID: 23917584
Note: Semi quantification
Source: Sci Rep. 2013;3:2364.

Dataset ID: M10
Taxon: Human
Omic: Metabolomics

Method: CE-MS
Region: Cerebrospinal fluid
Group (sample): 19 CN, 22 MCI, 9 MCI to AD, 23 AD
Criteria: forward stepwise LDA
PMID: 22967182
Note: Semi quantification
Source: Anal Chem. 2012 Oct 16;84(20):8532-40.

Dataset ID: M11
Taxon: Human
Omic: Metabolomics
Method: LC-MS
Region: Blood
Group (sample): 22 CN vs 22 EAD
Criteria: $p < 0.05$
PMID: 31488882
Note: Absolute quantification
Source: Cell Res. 2019 Oct;29(10):787-803.

Dataset ID: M12
Taxon: Human
Omic: Metabolomics
Method: GC-MS
Region: Serum
Group (sample): 23 CN vs 23 AD
Criteria: VIP > 1.5
PMID: 25575172
Note: Semi quantification
Source: J Pharm Biomed Anal. 2015 Mar 25;107:75-81.

Dataset ID: M13
Taxon: Human
Omic: Metabolomics
Method: Various. Download the raw data for more details.
Note: This dataset was retrieved from HMDB using the disease keyword "Alzheimer's disease".
Source: HMDB

Dataset ID: V1
Note: These genes were composed of amyloid seed genes, tauopathy seed genes; late-onset AD common risk genes identified by large-scale genetic studies, and high quailty disease gene integration.
Source: Literature

Dataset ID: V2

Note: These genes satisfied at least one of the following conditions: i) gene validation in large-scale amyloid GWAS studies; ii) in vivo experimental model evidence that knockdown or overexpression of the gene leads to AD-like amyloid pathology.
Source: Literature

Dataset ID: V3
Note: These genes satisfied at least one of the following conditions: i) gene validation in large-scale tauopathy GWAS studies; ii) in vivo experimental model evidence that knockdown or overexpression of the gene leads to AD-like tau pathology.
Source: Literature

Dataset ID: V4
Note: The 103 risk genes were predicted based on the hypothesis that the true risk genes are more densely linked with each other in a biological network. Nat Neurosci. 2019;22(5):691-699.
Source: Literature

Dataset ID: V5
Note: 404 genes co-occuring with the biological term Alzheimer in literature-supported statements describing functions of genes from the GeneRIF Biological Term Annotations dataset.
Source: GeneRIF-Biological-Term-Annotations

Dataset ID: V6
Note: 15 genes associated with Alzheimer's disease; Alzheimer's disease in GWAS and other genetic association datasets from the GAD Gene-Disease Associations dataset.
Source: GAD Gene-Disease Associations

Dataset ID: V7
Note: Alzheimer's Disease CNS - Brain - Hippocampus (MMHCC) GSE1297
Source: GEO Signatures of Differentially Expressed Genes for Diseases

Dataset ID: V8
Note: Alzheimer's Disease Entorhinal Cortex GSE5281
Source: GEO Signatures of Differentially Expressed Genes for Diseases

Dataset ID: V9
Note: 33 genes associated with Alzheimer's disease phenotype in GWAS datasets from the GWAS Catalog SNP-Phenotype Associations dataset.
Source: GWAS Catalog SNP-Phenotype Associations

Dataset ID: V10
Note: 34 genes associated with Alzheimer's disease (late onset) phenotype in GWAS datasets from the GWAS Catalog SNP-Phenotype Associations dataset.
Source: GWAS Catalog SNP-Phenotype Associations

Dataset ID: V11
Note: 41 genes associated with Alzheimer's disease (cognitive decline) phenotype in GWAS datasets from the GWAS Catalog SNP-Phenotype Associations dataset.
Source: GWAS Catalog SNP-Phenotype Associations

Dataset ID: V12
Note: 28 proteins participating in Alzheimer's disease pathway from the KEGG Pathways dataset.
Source: KEGG Pathways

Dataset ID: V13
Note: 55 proteins participating in Alzheimer's disease-amyloid secretase pathway from the PANTHER Pathways dataset.
Source: PANTHER Pathways

Dataset ID: V14
Note: 98 proteins participating in Alzheimer's disease-presenilin pathway from the PANTHER Pathways dataset.
Source: PANTHER Pathways

Dataset ID: V15
Note: 66 proteins co-occuring with the biological term Alzheimer in the abstracts of publications describing phosphosites from the Phosphosite Textmining Biological Term Annotations dataset.
Source: Phosphosite Text-mining Biological Term Annotations

Dataset ID: V16
Note: 226 proteins co-occuring with Alzheimer's disease specific cell type in the abstracts of biomedical publications from the TISSUES Text-mining Tissue Protein Expression Evidence Scores dataset.
Source: TISSUES Text-mining Tissue Protein Expression Evidence Scores

Dataset ID: V17
Note: 79 proteins participating in Alzheimers Disease (Homo sapiens) pathway from the Wikipathways Pathways dataset.
Source: Wikipathways Pathways

Dataset ID: V18
Note: 73 proteins participating in Alzheimers Disease (Mus musculus) pathway from the Wikipathways Pathways dataset.
Source: Wikipathways Pathways

Dataset ID: V19
Note: 17 proteins associated with Alzheimer's disease from the curated PhosphoSitePlus Phosphosite-Disease Associations dataset.
Source: PhosphoSitePlus Phosphosite-Disease Associations

Dataset ID: V20
Note: Overall score>0.3 and S_literature>0. Retrieve on Nov 18th, 2019.
Source: Open Targets

Dataset ID: V21
Note: C0002395, Score_gda>0.3. Retrieve on Nov 18th, 2019.
Source: DisGenet

Dataset ID: V22
Note: C0494463, Score_gda>0.3. Retrieve on Nov 18th, 2019.
Source: DisGenet

Dataset ID: V23
Note: C0750901, Score_gda>0.3. Retrieve on Nov 18th, 2019.
Source: DisGenet

Dataset ID: V24
Note: C0276496, Score_gda>0.3. Retrieve on Nov 18th, 2019.
Source: DisGenet

Dataset ID: V25
Note: Retrieve on Nov 18th, 2019.
Source: ClinVar

Dataset ID: V26
Note: All genes have at least 3 related publications. Retrieve on Nov 18th, 2019.
Source: Phenopedia

Dataset ID: V27
Note: D000544, excludinng the gene without direct evidence. Retrieve on Nov 18th, 2019.
Source: The Comparative Toxicogenomics Database (CTD)
